# Hand-Book of Materia Medica, Pharmacy and Therapeutics

**Published:** 1889-03

**Authors:** 


**Hand-Book of Materia Medica, Pharmacy and Therapeutics.** Compiled for the use of students preparing for examination. By CUTHBERT BOWEN, M. D., B. A., editor of "Notes on Practice." Pp. vi., 366. Philadelphia and London: F. A. Davis. 1888.

The hand-book, compiled for the use of students, should be considered from their standpoint. As such we can recommend it as reliable and comprehensive. The facts of materia medica and therapeutics have accumulated so greatly within the last years, that that department is often the most dreaded of any in the course of study of the medical student. Many of the text-books have grown so large, and include so much, that the student is lost in the attempt to understand the subject. On the other hand, we do not favor short-cuts to knowledge. Hard work is the only thing that counts for much in the pursuit of scientific medicine. Dr. Bowen's work seems to possess the merit of being complete and concise. It is arranged in the form of questions and answers, and under the latter are given examples of the subject considered. Then follows suggestions of the administration of the drug in prescriptions. In this way, it teaches prescription-writing, the art of combining medicines, incompatibility, the action and the indications of remedies.

The introductory chapter contains, among other things, the metric system, definition of terms, rules for dosage, classification of drugs, the administration and absorption, etc., of remedies, and other important subjects.

The book is carefully and well printed. On the whole, we can say that we know of no better work for the purpose for which it is intended.

